# Draft Genome Sequence of *Bacillus velezensis* OSY-S3, a Producer of Potent Antimicrobial Agents Active against Bacteria and Fungi

**DOI:** 10.1128/genomeA.01465-17

**Published:** 2018-01-18

**Authors:** Michelle M. Gerst, Mustafa Yesil, Ahmed E. Yousef

**Affiliations:** a Department of Microbiology, The Ohio State University, Columbus, Ohio, USA; b Department of Food Science and Technology, The Ohio State University, Columbus, Ohio, USA

## Abstract

*Bacillus velezensis* OSY-S3 produces anti-*Listeria*, anti-*Escherichia coli*, and antifungal compounds. Additionally, fermentate of *B. velezensis* OSY-S3 culture removes *Staphylococcus aureus* biofilms effectively. The draft genome sequence of *B. velezensis* OSY-S3 reported here had a genome size of ~3.90 Mb and a G+C content of 46.5%.

## GENOME ANNOUNCEMENT

*Bacillus* spp. are known to produce a multiplicity of functional metabolites, including antimicrobial compounds (1). *B. velezensis* OSY-S3, a strain isolated recently from silage, produced multifunctional antimicrobial agents. Fermentate of *B. velezensis* OSY-S3 culture inhibited *Listeria innocua* on agar plate and removed *Staphylococcus aureus* biofilms in 96-well plates (M. M. Gerst, unpublished data). Crude cell extract from *B. velezensis* OSY-S3 inhibited *L. innocua*, *Escherichia coli*, *Penicillium* sp., *Cladosporium* sp., and *S. aureus* (M. M. Gerst, unpublished data).

The whole genome of *B. velezensis* OSY-S3 was sequenced at The Ohio State University Nucleic Acid Shared Resource Facility (Columbus, OH, USA) using Ion Torrent next-generation sequencing technology. Raw data, in the form of 1,170,526 reads, were *de novo* assembled into 104 contigs using the SPAdes version 3.10.1 genome assembler (2). The contigs were ordered against *B. subtilis* 916 (reference genome) using progressiveMAUVE multiple genome alignment software (3), with one N representing each gap. The maximum contig size was 192,427 bp, and the minimum size was 206 bp. The NCBI Prokaryotic Genome Annotation Pipeline was used to annotate the *B. velezensis* OSY-S3 draft genome. The genome mining programs BAGEL3 and antiSMASH version 4.0.3 were used to identify secondary metabolite gene clusters (4, 5).

A total of 12 secondary metabolite gene clusters were identified using antiSMASH. Among these metabolites, antimicrobial agents with similarity to surfactin (91%), macrolactin (100%), bacillaene (100%), fengycin (100%), difficidin (100%), bacillibactin (100%), and bacilysin (100%) were recognized. Two additional antimicrobial peptides, plantazolicin and LCI, were identified using BAGEL3. Genes for many of these antimicrobial agents can also be found in in the genome of *B. velezensis* FZB42 (previously *B. amyloliquefaciens* subsp. *plantarum* FZB42) (6). Macrolactin, bacillaene, difficidin, bacilysin, and plantazolicin are known for activity against Gram-positive bacteria (7–11). Bacillaene, difficidin, and bacilysin are active against Gram-negative bacteria (7–9). Fengycin has antifungal activity (12). Sequencing the whole genome of *B. velezensis* OSY-S3 provided insight into the sources of the broad antimicrobial activity exhibited by this bacterium. The capability to produce several potent antimicrobial compounds makes *B. velezensis* OSY-S3 a candidate strain for combating pathogenic microorganisms in food, agriculture, and medicine.

### Accession number(s).

The *B. velezensis* OSY-S3 whole-genome sequence has been deposited in NCBI/GenBank under the accession no. CP024706. The version described in this paper is the final version.

## References

[1] Kadaikunnan S, Rejiniemon T, Khaled JM, Alharbi NS, Mothana R 2015 *In-vitro* antibacterial, antifungal, antioxidant and functional properties of *Bacillus amyloliquefaciens*. Ann Clin Microbiol Antimicrob 14:9. doi:10.1186/s12941-015-0069-1. 25858278 PMC4342198

[2] Bankevich A, Nurk S, Antipov D, Gurevich AA, Dvorkin M, Kulikov AS, Lesin VM, Nikolenko SI, Pham S, Prjibelski AD, Pyshkin AV, Sirotkin AV, Vyahhi N, Tesler G, Alekseyev MA, Pevzner PA 2012 SPAdes: a new genome assembly algorithm and its applications to single-cell sequencing. J Comput Biol 19:455–477. doi:10.1089/cmb.2012.0021. 22506599 PMC3342519

[3] Rissman AI, Mau B, Biehl BS, Darling AE, Glasner JD, Perna NT 2009 Reordering contigs of draft genomes using the Mauve aligner. Bioinformatics 25:2071–2073. doi:10.1093/bioinformatics/btp356. 19515959 PMC2723005

[4] van Heel AJ, de Jong A, Montalbán-López M, Kok J, Kuipers OP 2013 BAGEL3: automated identification of genes encoding bacteriocins and (non-)bactericidal posttranslationally modified peptides. Nucleic Acids Res 41:W448–W453. doi:10.1093/nar/gkt391. 23677608 PMC3692055

[5] Blin K, Wolf T, Chevrette MG, Lu X, Schwalen CJ, Kautsar SA, Suarez Duran HG, de los Santos ELC, Kim HU, Nave M, Dickschat JS, Mitchell DA, Shelest E, Breitling R, Takano E, Lee SY, Weber T, Medema MH 2017 antiSMASH 4.0—improvements in chemistry prediction and gene cluster boundary identification. Nucleic Acids Res 45:W36–W41. doi:10.1093/nar/gkx319. PMC5570095 28460038

[6] Chowdhury SP, Hartmann A, Gao X, Borriss R 2015 Biocontrol mechanism by root-associated *Bacillus amyloliquefaciens* FZB42—a review. Front Microbiol 6:780. doi:10.3389/fmicb.2015.00780. 26284057 PMC4517070

[7] Jaruchoktaweechai C, Suwanborirux K, Tanasupawatt S, Kittakoop P, Menasveta P 2000 New macrolactins from a marine *Bacillus* sp. Sc026. J Nat Prod 63:984–986. doi:10.1021/np990605c. 10924180

[8] Patel PS, Huang S, Fisher S, Pirnik D, Aklonis C, Dean L, Meyers E, Fernandes P, Mayerl F 1995 Bacillaene, a novel inhibitor of prokaryotic protein synthesis produced by *Bacillus subtilis*: production, taxonomy, isolation, physico-chemical characterization and biological activity. J Antibiot 48:997–1003. doi:10.7164/antibiotics.48.997. 7592068

[9] Wilson KE, Flor JE, Schwartz RE, Joshua H, Smith JL, Pelak BA, Liesch JM, Hensens OD 1987 Difficidin and oxydifficidin: novel broad spectrum antibacterial antibiotics produced by *Bacillus subtilis*. II. Isolation and physico-chemical characterization. J Antibiot (Tokyo) 40:1682–1691. doi:10.7164/antibiotics.40.1682. 3429336

[10] Kenig M, Abraham EP 1976 Antimicrobial activities and antagonists of bacilysin and anticapsin. J Gen Microbiol 94:37–45. doi:10.1099/00221287-94-1-37. 819623

[11] Scholz R, Molohon KJ, Nachtigall J, Vater J, Markley AL, Süssmuth RD, Mitchell DA, Borriss R 2011 Plantazolicin, a novel microcin B17/streptolysin S-like natural product from *Bacillus amyloliquefaciens* FZB42. J Bacteriol 193:215–224. doi:10.1128/JB.00784-10. 20971906 PMC3019963

[12] Vanittanakom N, Loeffler W, Koch U, Jung G 1986 Fengycin—a novel antifungal lipopeptide antibiotic produced by *Bacillus subtilis* F-29-3. J Antibiot (Tokyo) 39:888–901. doi:10.7164/antibiotics.39.888. 3093430

